# Application of Radiomics and Decision Support Systems for Breast MR Differential Diagnosis

**DOI:** 10.1155/2018/7417126

**Published:** 2018-09-23

**Authors:** Ioannis Tsougos, Alexandros Vamvakas, Constantin Kappas, Ioannis Fezoulidis, Katerina Vassiou

**Affiliations:** ^1^Medical Physics Department, Medical School, University of Thessaly, Larissa, Greece; ^2^Radiology Department, Medical School, University of Thessaly, Larissa, Greece; ^3^Anatomy Department, Medical School, University of Thessaly, Larissa, Greece

## Abstract

Over the years, MR systems have evolved from imaging modalities to advanced computational systems producing a variety of numerical parameters that can be used for the noninvasive preoperative assessment of breast pathology. Furthermore, the combination with state-of-the-art image analysis methods provides a plethora of quantifiable imaging features, termed radiomics that increases diagnostic accuracy towards individualized therapy planning. More importantly, radiomics can now be complemented by the emerging deep learning techniques for further process automation and correlation with other clinical data which facilitate the monitoring of treatment response, as well as the prediction of patient's outcome, by means of unravelling of the complex underlying pathophysiological mechanisms which are reflected in tissue phenotype. The scope of this review is to provide applications and limitations of radiomics towards the development of clinical decision support systems for breast cancer diagnosis and prognosis.

## 1. Introduction

Breast cancer is the most common female cancer and the first cause of cancer death in women population. Breast cancer is categorized into benign and malignant lesions. Benign lesions consist of noncancerous cells, including intralobular papillomas, (fibro/neuro) adenomas, phyllodes tumors, inflammatory tumors, cysts, etc. Malignant breast lesions are characterized as invasive or noninvasive, depending on the tumor corresponding growth pattern. Generally, invasive lesions are more aggressive and spread outside the breast. On the contrary, noninvasive breast lesions do not exceed breast tissue boundaries, and they are also known as carcinomas in situ. Also breast lesions are divided into ductal or lobular, considering the primary breast's anatomical region of cancer cell's arisen, i.e., ducts or lobules. In summary, the four major malignant breast lesion categories are invasive ductal carcinomas (IDCs), invasive lobular carcinomas (ILCs), ductal carcinomas in situ (DCIS), and lobular carcinomas in situ (LCIS). Invasive ductal carcinomas (IDCs) are the most common type of breast cancer with many thousand new cases diagnosed every year.

Nowadays, the so-called personalized approach to medical care is based on the large-scale data synthesis from different sources, such as the new generation molecular biology “-omics” tools (e.g., genomics (DNA), proteomics (proteins), metabolomics (metabolites), and transcriptomics (RNA)), as well as other factors (heredity and lifestyle) so that a holistic description of the pathology for each patient is created. The ultimate goal of this process is to classify patients into subgroups with common biological characteristics (e.g., expression of specific genes or predicted response to treatment), hypothesizing that different population groups may present different susceptibility to disease, and as a result, a requirement for more specialized diagnostic, prognostic, and therapeutic tools. Especially in cancerous tumors, the very important contribution of the above technologies, which aim at characterizing the biological heterogeneity of the lesions, by identifying the molecular phenotypes of the mutations of the disease, is already evident [1].

However, malignant tumor spatiotemporal biological heterogeneity is prone to underestimation errors as all these techniques are usually based on the analysis of small invasive biopsy tissue samples, hence presenting limitations in mapping the biological variations within the whole tumor site [2]. Nevertheless, it has to be noted that whole-mount breast biopsy methods have also been adapted in the clinical practice; however, there is still a need for utilizing large size tissue sample for techniques validation, as well as minimizing the cost-effectiveness for assessing the histopathology in the future [3].

Towards this direction, medical imaging has become a very valuable component of medical science contributing to the assessment of a variety of pathological conditions. Especially magnetic resonance imaging (MRI) has now evolved from simple imaging modality to an advanced computational system producing a variety of numerical parameters and can be used for the noninvasive preoperative assessment of pathology [4]. In terms of personalized cancer treatment, advanced imaging analysis aims at the unraveling of tumor heterogeneity, guiding cancer diagnosis, staging and planning interventions for treating patients, monitoring therapeutic approaches, predicting treatment response, and determining outcomes [5].

In addition, this promising field of research based on the quantitative study of imaging phenotypes, termed “Radiomics”, has been integrated to clinical practice for breast diagnosis and treatment management.

## 2. Breast Imaging and MRI

In clinical routine, several imaging modalities for breast cancer screening prior to treatment are used, including mammography, ultrasonography (US), digital breast tomosynthesis (DBT), and magnetic resonance imaging (MRI). MRI holds a leading role in screening the particular group of high-risk women, due to its advantages regarding spatial resolution, generation of high-contrast images for soft tissue, and the lack of ionizing radiation. Nevertheless, conventional MR imaging findings in a number of breast masses are sometimes nonspecific, and despite the recent technological advancements, breast tumor diagnosis (detection or characterization) and prognosis (response to therapy, morbidity, and mortality) can be very challenging processes [6]. Therefore, continual efforts are being made to assess the utility of advanced MR imaging techniques and enable the extraction of quantifiable features for the assessment of malignant breast tumors aggressiveness in a reliable manner.

The parameters extracted from the various advanced MR imaging techniques, such as diffusion weighted/tensor imaging (DWI/DTI), perfusion weighted imaging (PWI), MR spectroscopy (MRS), and MR elastography (MRE), provide significant structural and functional information in a microscopic and cellular level, highlighting aspects of the underlying breast pathophysiology, regarding cellularity, neovascularization, and tumor biochemical processes. Usually, the most critical elements in the determination of tumor grade and prognosis are tumor cellularity and vascularity. These elements can be quantified using diffusion and perfusion techniques, respectively.

In the past decade, numerous studies have reported the value of DWI for differentiation between benign and malignant breast tumors, by means of apparent diffusion coefficient (ADC) parametric maps [7–9]. Choi et al. [10] showed that malignant tumors have decreased the ADC value proportional to their increased cellularity. However, the same study concludes that lower ADC values of malignant tumors may be due to tumor angiogenesis, as DWI parameters are influenced by perfusion effects. In addition, recent studies have supported the correlation of ADC values with several prognostic factors of biological markers, including ER, PR, HER2, and Ki-67 statuses [11–13].

Perfusion weighted MR imaging, on the contrary, presents an equivalent contribution by means of assessing tumor angiogenesis, vascularity, and vessel permeability mainly utilizing dynamic contrast-enhanced (DCE) imaging techniques. The high gadolinium uptake by tumors helps the clinicians in the accurate differentiation of breast lesions compared to normal tissue. Furthermore, DCE-MRI signal time-series evaluation through empirical or pharmacokinetic models has presented robust results for tumor characterization [14, 15], as well as monitoring response to therapy parameters [16, 17]. For example, benign and malignant breast lesions differ in the characteristics of their microvessels, and hence in their behavior of gadolinium uptake in the lesion which can be measured with the pharmacokinetic parameters of vascular permeability, such as the transfer constant *K*^trans^, *K*^trans^ measures the transit of contrast agent through the vascular bed at the capillary level and reflects qualitative changes of tumor vessels (i.e., increased porosity/permeability), a surrogate of neoangiogenesis. In addition to *K*^trans^, several tissue specific kinetic parameters may be estimated including the volume fraction of the extravascular extracellular space (*v*_e_) in tissue, the volume fraction of plasma in tissue (*v*_p_), and the rate constant for efflux of gadolinium contrast back into plasma from the tissue extracellular space (*k*_ep_) [14].

In addition, in vivo 1H-MRS is a noninvasive method for characterizing the cellular biochemistry which underlies breast pathologies, by monitoring the choline concentration. As choline complexes are believed to be precursors of the phospholipids that compose cell membranes, increases in choline signals are thought to reflect increased membrane synthesis. It can be considered as a bridge between metabolism and the anatomic and physiological studies available from MRI. Breast 1H-MRS is proposed to be used as an adjunct tool to MRI examination for the improvement of specificity. 1H-MRS can provide a qualitative and/or a quantitative analysis of a number of metabolites within the tissue under study [18–21].

Nevertheless, tumor biological processes are closely correlated; their accurate interpretation is not always straightforward and becomes difficult on the basis of individual numeric parameters, as similarities may exist between pathologies, and one should be very careful in correctly evaluating all available MR data [22]. Recently, multiparametric approaches are proposed for improving the diagnostic accuracy through the correlation of the results between different MR imaging techniques [23–25].

At this point however, it should also be realized that the vast amounts of data and numerical parameters produced by the advanced MRI techniques may pose more of a problem rather than a solution. Why? Because the endogenous complexity of the sophisticated imaging methods and big datasets, as well as the nature of the imaging features, trouble radiologists in collecting and rationalizing the abundance of these important quantitative metrics, as well as accurately evaluating them during the clinical routine.

Hence, despite the indisputable, contribution of advanced techniques to the preoperative assessment of breast pathology, the often-contradictory character of individual diagnostic results, which is a consequence of the complex biological correlations they reflect, requires the overcoming of the classical qualitative assessment methods. For that reason, the advances in information technology have led state-of-the-art image analysis methods to be implemented in the field of medical imaging, in terms of improving and managing the diagnostic outcome, introducing the rapidly evolving field of radiomics.

## 3. Radiomics and Decision Support in Breast MRI

The development of radiomics, which is the conversion of medical images into quantifiable data which facilitate the clinical decision support for improving diagnostic, prognostic, and predictive accuracy, is motivated by the concept that images are more than pictures and contain valuable information about the tissue underlying pathophysiological characteristics, which can be extracted with advanced computational tools [5, 6, 26].

Radiomics can be considered as an extension of computer-aided diagnosis (CAD) systems which have been successfully used until recently, especially applied in breast cancer detection (CADe) and diagnosis (CADx) [26]. In fact, the feasibility for implementing automated techniques for breast cancer imaging, by means of CAD systems, arises from the challenging clinical questions regarding lesion delineation from breast's diffusive parenchyma (e.g., detection of masses, microcalcifications, and architectural distortions), as also breast lesion biological characterization (e.g., benign vs malignant, tumor grading, and BIRADS categorization).

More specifically, CAD algorithms are composed of two stages, that is, detection and classification of suspicious regions into cancerous and normal tissues. Firstly, detection is performed using basic image enhancement methods, descriptors of statistical distribution of intensity values, and decomposition of the image through wavelet transforms, in order to investigate differences between tumorous areas and background. Subsequently, CAD systems use a set of quantitative image features describing the geometrical structure, intensity distribution, and texture of a region of interest (ROI), automatically or manually contoured. Since many features can be extracted, CAD systems frequently incorporate feature selection algorithms to obtain the features contributing the most to diagnostic accuracy. Finally, the abovementioned systems may include statistical or machine learning classifiers in order to distinguish cancerous lesions from normal breast tissue [27].

Therefore, CAD systems constitute supplementary tools to radiologists, for evaluating the results from different imaging modalities, towards detecting lesions and making diagnostic decisions.

Obviously CAD can be considered part of radiomics, but in contrast to CAD's simplicity and ability for answering only simple clinical questions, radiomic analysis considers more complex computational processes aiding decision support, by utilizing a plethora of quantitative imaging features—potential imaging biomarkers, extracted from digital images [26, 28]. Furthermore, the correlation of these large-scale radiological phenotypic characteristics with the rich breast histopathological data available, e.g., the expression statuses of estrogen receptor (ER), progesterone receptor (PR), human epidermal growth factor 2 receptor (HER2), and triple negative (lack of expression of ER, PR, and HER2), facilitates their strong association with molecular subtypes, which eventually results in the generation of pathology prognostic and predictive models [4, 26, 27, 29].

The central hypothesis of radiomic analysis is that these libraries of quantitative individual voxel-based variables are more sensitively associated with various clinical endpoints compared with the more qualitative radiologic, histopathologic, and clinical data more commonly utilized today [30], especially taking into account that the aforementioned key information originating from routine clinical imaging usually remains unexploited.

Therefore, the knowledge about these deep biological mechanisms which are reflected into tissue phenotype, obtained from radiomic analysis, and potentially enhanced by the combination with other -omics (e.g., radiogenomics [31]), is a very important step towards individualized therapy planning and building of models for predicting patient outcome [5].

However, radiomic analysis is still a very challenging process, facing complex technical difficulties (medical data collection and development of novel computational methods) and methodological challenges (poor study design, data overfitting, and lack of standards for results validating), and will be analyzed below [6, 32].

## 4. Radiomics Analysis Workflow

### 4.1. Segmentation

Image segmentation is usually the first step, after data preprocessing (noise reduction, correction of artifacts, normalization, etc.), in the radiomic analysis workflow towards lesion evaluation for diagnosis and selection of appropriate treatment plan. The precise definition of breast lesion boundaries is a very important procedure, as it affects the subsequent qualitative analysis of the radiomic descriptors extracted from the corresponding regions or volumes of interest (ROI/VOI). In daily clinical routine, ROIs are manually segmented by expert radiologists, but besides its time-consuming nature, this approach induces intra-/interobserver variability and reproducibility errors, as many tumors present indistinct and blurring boundaries [33]. The development and validation of novel semiautomated or automated segmentation algorithms is an open research field which presents interesting and sophisticated results. However, the semiautomated approaches are mandatory so that the final choice remains user-dependent since fully automated methods are feasible only if there are strong signal differences between the lesion and the background [34]. In addition, time-cost minimization for segmenting all tumor slices in tomographic imaging modalities, such as MRI, enables the reconstruction of three-dimensional (3D) tumor models, which further facilitate the global assessment of the pathology.

The initial approaches towards automated breast tumor delineation methods for CAD systems included intensity-based methods utilizing histogram thresholding for edge enhancement in mammographic data [35]. However, the main disadvantage of the thresholding methods is the spatial incoherence (scattering) presented in segmented regions, as this method does not take into account pixels' neighborhood information. Region growing methods are an evolution, where segment's coherence is obtained from application of conditions by the user, such as homogeneity criteria between neighboring voxels and mostly the inclusion of manually induced seed pixels in the final segment [36]. Classification and clustering methods have been developed for classifying image pixels into different groups of similar intensities, thus properties, utilizing sophisticated algorithms such as *k*-NN, *k*-means, and fuzzy *c*-means [37–40]. Furthermore, this kind of segmentation method performed on fused MR images (T1, T2, DWI, and DCE) enables tumor separation into subregions (habitat imaging), which contributes in the revelation of tumor heterogeneity and potential selected region-based feature extraction for adaptive analyses [41]. Lately, several free and widely accepted software packages exist providing semiautomated segmentation tools, based on a variety of algorithms (e.g., watershed and active contours/surfaces), such as 3DSlicer (www.slicer.org) and ITK-SNAP (www.itksnap.org/).

### 4.2. Radiomic Descriptors

After tumor delineation, radiomic features are extracted from the information contained in the segmented ROIs that can be used to qualitatively assess tumor phenotype, aggressiveness, treatment response, and cancer genetics, and differentiate between benign and malignant tumors [5]. The further processing and selection between the varieties of radiomic features derived leads to the potential definition of qualitative imaging biomarkers (QIB) that holds prognostic and predictive values for cancer outcome [28]. Therefore, when found to have significant correlation with tumor's biological properties, these parameters may possibly serve as useful endpoints for the assessment of the severity, degree of change, or status of a cancer lesion, relative to normal [22].

Besides various metrics derived from advanced MR techniques, novel approaches such as texture analysis seems to overcome limitations regarding diagnostic accuracy and reproducibility [42]. Texture features provide more detailed structural and dimensional information of pixel intensity values distribution, which facilitates a more effective intercomparison between images by means of the upgraded quantitative perception of tissue imaging characteristics.

Radiomic features may be divided into several categories depending on their characteristics, such as shape- and size-based, histogram-based, textural and transform-based features [2, 27]. Shape- and size-based features provide information about tumor location and different size parameters, like surface, volume, diameter, sphericity, and surface-to-volume ratio. First-order histogram parameters, such as mean value, standard deviation, percentiles, skewness, kurtosis, and entropy, enable the rough assessment of pixel intensity global distribution without considering spatial variations. Different reported studies supported that histogram analysis of ADC parametric maps in breast DWI may serve as a prognostic biomarker [10, 13].

Higher order statistics derived features, referred to as textural features, have been widely utilized in breast tumor DCE-MRI parametric maps in the past, for improving characterization of breast lesions and their response to treatment [43–47]. Second-order histograms such as gray-level co-occurrence matrices (GLCMs) [28, 48] and gray-level run-length matrices (GLRLMs) [29, 49] characterize spatial relationships between pixel intensities in different 2D or 3D directions and thus are robust in quantifying tumor structural properties and various patterns of heterogeneity. In particular, GLCM analysis of DCE MR data has been proved to be robust in differentiating between benign and malignant breast lesions [50]. Finally, additional high-order textural features, such as Gabor textures, temporal kinetics, and fractal-based textures, have been employed for classifying malignant from benign breast tumors, by means of identifying texture-related signal variations [51, 52].

To date, several studies have focused on the correlation and integration of radiomic features with breast genomics and proteomics data, and their results continue to support the notion that radiomic metrics may perform well for better understanding of molecular and genetic variability, protein expression and predicting prognosis, and response to therapy [41, 53]. Considering breast tumors, recent studies have reported the potential prediction of tumor subtypes [54], as well as clinical phenotypes through the association of breast tumor MR imaging data with ER, PR, and HER2 statuses [55].

In the past, various reliable texture analysis software tools have been developed, such as the open access MaZda (http://www.eletel.p.lodz.pl/programy/mazda/) and the commercially available TexRAD (http://texrad.com/).

## 5. Pattern Recognition and Decision Support

Despite the indisputable contribution of the advanced MR techniques and image analysis methods to the preoperative assessment of breast tumors, unfortunately, the immense numbers of imaging and clinical features involved still challenge current methods of qualitative analysis. On the contrary, data analysis using conventional methods such as statistical significances correlations of the related parameters between different tumor groups may be efficient in a limited number of cases. However, in more demanding diagnostic problems like pathologies mimicking tumors or lesions with identical pathophysiological profiles, where data ranges are overlapping, the statistically significant correlations might be limited [22]. Thus, innovative computer-assisted diagnostic tools are required to analyze multidimensional data and to illustrate the above relationships in intelligible and measurable quantities.

In the past, several advanced methods of data analysis have been evaluated, such as logistic regression (LR) [56, 57] and Bayesian classifiers [58] aiming in the incremental of MRI diagnostic accuracy. However, the abovementioned computational processes proved quite demanding and time-consuming and moreover presented limitations in evaluating the big amount of radiomic descriptors generated.

It is interesting to note that recently, research and clinical interest have been focused on the incremental of diagnostic and predictive value of breast MR multiparametric approaches using advanced machine learning classifiers, like support vector machine (SVM) and *k*-nearest neighbor (*k-*NN) classifiers [58–62]. These techniques present huge potential to improve the understanding of complex pathological conditions, through identifying robust associations between morphological or functional changes in imaging and clinical variants linked to the diseases. Therefore, this holistic imaging biomarker-based description of the pathology, also called radiomic signature [62, 63], can be used towards the personalized patient care.

Machine learning techniques have been widely utilized for overcoming the limitations of data unilateral evaluation approaches, which are usually not robust enough to be used for high-precision diagnostic outcomes. Based on their ability to learn information from the provided training datasets, these sophisticated algorithms have demonstrated a superior efficiency in making accurate classifications of the features extracted from radiological images, achieving higher diagnostic accuracy of multiparametric MRI [62, 64].

Recent research studies have also reported that the implementation of deep learning classification techniques, such as artificial neural networks (ANNs), may be used as an automated computer analysis tool providing further process automation, in order to aid radiomic analysis with potential application to breast tumor diagnosis [65].

Deep learning, a class of machine learning algorithms, performs supervised classification tasks mimicking the way that the nervous system defines relationships between various stimuli and corresponding neuronal responses. It is an emerging field of data science, getting attention due to its promising applications in various science fields, as it has been shown to excel at learning a hierarchy of increasingly complex imaging features directly from raw data. Hence, it can be considered the alternative to the quite demanding and time-consuming conventional machine learning methods which involve segmentation, feature extraction, and classification steps. [66].

The use of such techniques in medical imaging allows the manipulation and evaluation of a large amount of quantitative data (also called big data) during clinical practice. Also, except for computational cost minimization, the main advantage of implementing deep learning classification schemes over radiomics workflow is their ability for extracting a large number of self-taught features in a totally automated way from raw imaging data. In addition, in two recent studies, Dalmis et al [67, 68] have shown the value of convolutional neural networks (CNN) and U-net for automated segmenting of breast lesions and whole breast and fibroglandular tissue, respectively.

However, the most important aspect of deep learning utilization is its advantage in performing multiparametric clinical big data associations (e.g., radiogenomic data), which facilitates the provision of pathology predictive outcomes and the development of intelligent clinical decision support systems (CDSS), for implementing individualized patient-specific diagnosis and prognosis approaches. Therefore, diagnostic and predictive results ascending from complex correlations will potentially accelerate the process of directly characterizing the aggressiveness of disease in an accurate way, leading to the robust evaluation and individual treatment planning of the pathology, in contrast with conventional statistical methods that are limited to producing diagnostic results retrospectively.

## 6. Limitations

Although the future of radiomic analysis seems promising, to date there are several limitations and challenges that must be overcome, mainly related to technical complexities in different aspects of the radiomic workflow. More specifically, radiomic features quantification is very sensitive to data acquisition parameters (medical image artifacts, reconstruction methods, and sampling) and variations of feature extraction methods [69]. Also, several limitations are related to lesion segmentation and feature extraction algorithms in terms of user dependency; thus the subjective selection of initial criteria finally affect accuracy, stability, and reproducibility of the proposed methods.

However, the main drawback of radiomics remains that the link between the imaged properties of tumors, and tumor biology is not straightforward; even though most radiomics studies show statistical correlation between radiomic features and genetic phenotype, this correlation does not imply causation [22]. Hence, there is a need for further systematic studies with proper design for results validation and establishment of standards.

## 7. Conclusion

**Figure 1:**
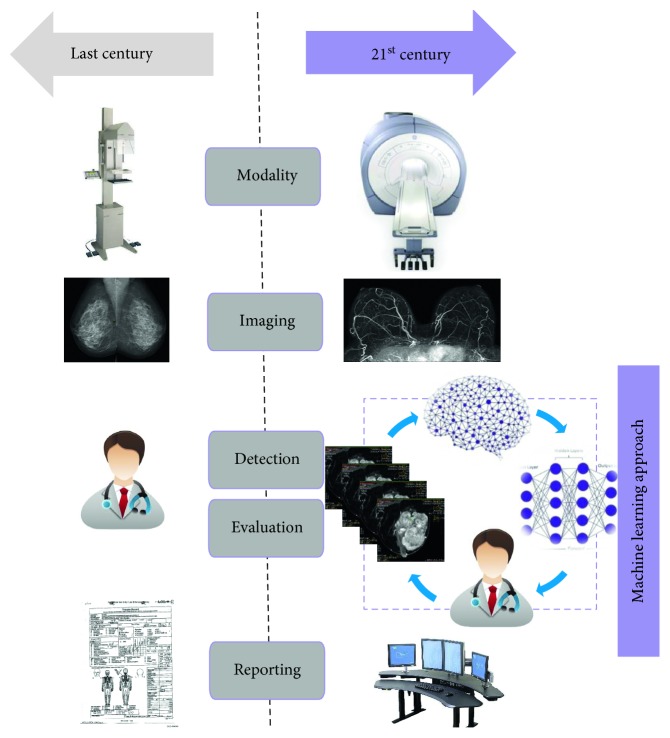
The evolution of breast medical imaging taking advantage of the new powerful modalities and advanced techniques, such as MRI, as well as the promising era of a machine learning approach towards the individualization of medical care and precision oncology.

In conclusion, it seems that the field of biomarker discovery has evolved rapidly over the past few years aiming to individualize medical care with personalized diagnosis and prognosis towards precision oncology.  Figure 1 is an attempt to evidentiary describe the “where do we stand?” rhetoric question arising from this review paper, specifically for breast cancer. It is evident that with the dawn of 21^st^ century, breast medical imaging has evolved, taking advantage of the new powerful modalities and advanced techniques such as MRI, as well as the promising era of implementing machine learning techniques in medical imaging. Obviously, the key to diagnosis and prognosis of breast tumors lies in a multiparametric evaluation scheme combining radiomics and biomarker analysis. Therefore, there is a need to utilize sophisticated computational methods in the clinical routine in order to develop and standardize specialized management and quantitative assessment procedures to maximize the diagnostic benefit (early detection, prognosis, and differential diagnosis) and to integrate the individualized medical act into the more general context.
